# Progressive Right Ventricular Obstruction Caused by a Double-Chambered Right Ventricle Resulting in Shunt-Reversal via a Concomitant Congenital Ventricular Septal Defect and Subsequent Erythrocytosis in a Dog

**DOI:** 10.3390/vetsci10030174

**Published:** 2023-02-21

**Authors:** Viktor Szatmári, Mark Dirven, Heike Aupperle-Lellbach

**Affiliations:** 1 Clinical Sciences, Faculty of Veterinary Medicine, Utrecht University, 3584CM Utrecht, The Netherlands; 2 Laboklin GMBH & CO.KG, 97688 Bad Kissingen, Germany

**Keywords:** cyanosis, endothelial-to-mesenchymal transition, Gasul phenomenon, endocardial fibrosis, hypoxemia, murmur, polycythemia, right-to-left shunt, shear stress, syncope

## Abstract

**Simple Summary:**

A 3-year-old Chihuahua was presented because of exercise intolerance, respiratory distress, and collapsing episodes. The dog was known to have a congenital heart disease, a ventricular septal defect. This anomaly was diagnosed using echocardiography at 10 weeks of age when the dog showed no clinical signs but had a murmur. The defect was considered unimportant then because only a small amount of blood flowed from the left to the right ventricle. However, at 3 years of age, a repeated echocardiogram revealed shunt reversal via the ventricular septal defect, allowing deoxygenated blood from the right ventricle to enter the systemic arteries, resulting in insufficient oxygen supply to all organs. The reason why the direction of blood flow through the ventricular septal defect reversed was the development of an obstructive band of tissue in the right ventricle, which caused the right ventricular pressure to exceed the left ventricular pressure. The most likely cause for the development of the abnormal band was thought to be the continuous mechanical irritation of the right ventricular wall caused by the high-velocity jet arising from the left ventricle. Because of the poor prognosis, the dog was euthanized, and a post-mortem examination took place.

**Abstract:**

A 3-year-old Chihuahua was presented because of exercise intolerance, respiratory distress, and syncopal episodes. At the age of 10 weeks, the dog was diagnosed with a congenital small left-to-right shunting ventricular septal defect and a mild right ventricular outflow tract obstruction via echocardiography. At that time, the dog was asymptomatic, but the breeder’s veterinarian heard a murmur. Both cardiac defects were judged to be clinically non-relevant at that time. However, at 3 years of age, echocardiography revealed a severe right ventricular obstruction, known as a double-chambered right ventricle, along with right-to-left shunting via the ventricular septal defect. Because of chronic hypoxemia due to the right-to-left shunting, erythrocytosis developed. Flow reversal via the shunt was caused by a progressively worsening right ventricular obstruction leading to a supra-systemic right ventricular systolic pressure. Because of the poor prognosis, the dog was euthanized, and the heart was submitted for post-mortem examination. Gross pathologic findings revealed the close proximity of the right ventricular obstructive lesion to the ventricular septal defect. Histopathology revealed localized muscular hypertrophy and severe endocardial fibrosis. The suspected pathogenesis of the progressive obstruction was infiltrative myocardial fibrosis due to turbulent blood flow from the left-to-right shunting ventricular septal defect, as described in humans.

## 1. Introduction

Ventricular septal defect (VSD) is a congenital heart disease characterized by an opening between the ventricles, allowing blood to shunt from one ventricle to the other in systole [1,2,3]. The direction of blood flow is determined by the size of the defect and the ventricular pressures. Small to moderate-sized VSDs, in the absence of other cardiac anomalies, typically allow for left-to-right shunting [1,2,3]. 

Double-chambered right ventricle (DCRV) is characterized by a discrete muscular band and/or fibrous tissue within the right ventricular chamber, leading to abnormal septation and an obstruction to blood flow [4,5,6]. Although DCRV is often listed among congenital heart diseases, it is more of a development anomaly with a congenital substrate, similar to subaortic stenosis [4,5,6]. Though VSD is among the five most common congenital heart diseases both in dogs and humans, DCRV is rare in both species [5,6,7,8,9]. Interestingly, the simultaneous presence of a DCRV and a VSD in the same heart is relatively common, where the high-velocity jet resulting from the left-to-right shunting VSD-flow is thought to lead to the tissue proliferation in the right ventricle, contributing to the development of a DCRV (also known as Gasul phenomenon) and a progressive right ventricular outflow tract obstruction [7,8,9]. Depending on the severity of the obstruction and the anatomical localization of the VSD, the obstruction caused by DCRV can increase the right ventricular pressure to such a degree that a supra-systemic systolic pressure develops, and flow reversal via the VSD takes place, resulting in an intracardiac right-to-left shunting [1,2,3,4,5,6,7,8,9,10,11,12]. A right-to-left shunting VSD can cause hypoxemia and on long-term also erythrocytosis because of increased renal erythropoietin production [13]. Associated clinical signs of hypoxemia are exercise intolerance, exertional dyspnea, and collapse, where erythrocytosis can exaggerate these problems [9,13].

The present case report is unique because, in this dog, the evolution of the disease process was documented from a left-to-right shunting VSD with a mild right ventricular obstruction to a right-to-left shunting VSD as a result of a severe right ventricular obstruction due to a DCRV. In addition, post-mortem examination allowed further macro- and microscopic evaluation of the right ventricular lesion, which revealed similar changes to those described in humans with DCRV.

## 2. Case Presentation

A 3-year-old female Chihuahua of 2.4 kg was presented to the cardiology service because of progressively worsening exertional dyspnea for a month’s duration and recent syncopal episodes. 

**Figure 1:**
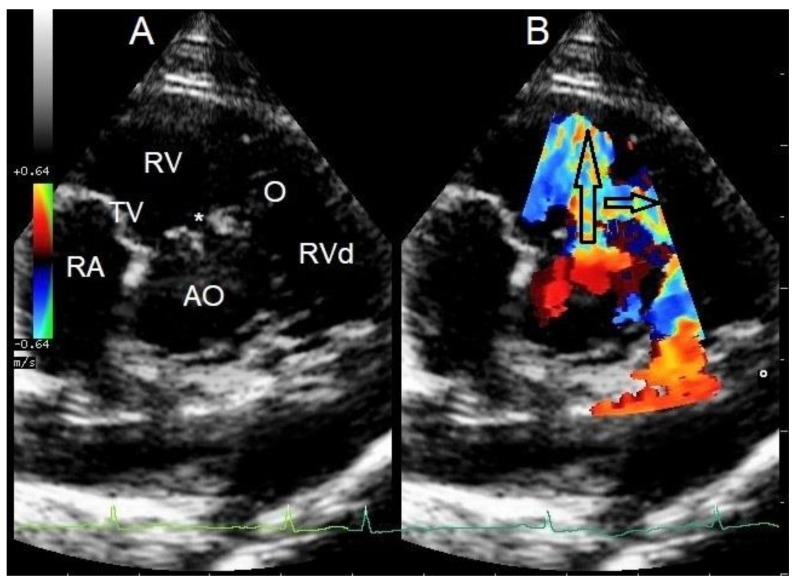
Cross-sectional echocardiographic images at the level of the heart base from the standard right parasternal short axis view performed at 10 weeks of age. (**A**) Two-dimensional image shows a small ventricular septal defect (*) close to the aortic (AO) and tricuspid valves (TV) and a localized muscular band in the right ventricle (O) just distal to the ventricular septal defect. RA—right atrium, RV—right ventricular chamber proximal to the obstruction, RVd—right ventricular chamber distal to the obstruction (**B**) Color Doppler image of the same view as in Figure 1A shows two high-velocity jets in the right ventricular chamber. The jet indicated by the large, vertically positioned arrow is caused by the left-to-right shunting ventricular septal defect, and the jet indicated by the smaller, horizontally positioned arrow is caused by the right ventricular intraluminal obstruction (O). The arrows also indicate the origin and direction of abnormal blood flow.

The breeder’s veterinarian found a murmur at a screening health examination at 10 weeks of age and referred the pup to a cardiologist for murmur evaluation. The puppy was free of clinical signs at that time. The cardiologist documented a systolic murmur with the point of maximal intensity on the right hemithorax and a murmur intensity of 5 out of 6. Echocardiography revealed a congenital small perimembranous left-to-right shunting VSD just underneath the aortic valve and with the right ventricular opening adjacent to the tricuspid valve (Figure 1). No echocardiographic signs of left ventricular volume overload or pulmonary hypertension were noted. In addition, localized muscular hypertrophy was noticed just distal to the VSD in the right ventricle. Continuous wave Doppler interrogation of blood flow through the VSD revealed left-to-right shunting with an estimated pressure difference between the ventricles of 64 mmHg. Continuous wave Doppler interrogation of the right ventricular outflow tract over the right ventricular obstructive lesion revealed a pressure gradient of 30 mmHg. The muscular band divided the right ventricle in a high-pressure and a normal-pressure compartment. The VSD connected the left ventricle to the high-pressure compartment of the right ventricle. Normally, no pressure gradient would be measured in the right ventricle. A left-to-right shunting restrictive VSD without right-sided pathology would typically be associated with a pressure gradient of 80–120 mmHg [1,2,3].

Because of the presenting problems at 3 years of age, the referring veterinarian suspected congestive left-sided heart failure and started oral furosemide and pimobendan administration four days prior to referral. This therapy resulted in some improvement of the dyspnea, according to the owner.

At presentation to the cardiology service at the authors’ institution, a physical examination revealed a bright, alert, and responsive dog with a body condition score of 4 out of 9. No signs of respiratory distress were apparent. Respiratory rate was 28 breaths/minute with a costo-abdominal breathing pattern. The femoral pulses were strong, regular, and symmetric, with a rate of 88/minute, without pulse deficit. The rectal temperature was 38.7 degrees of Celsius. The mucous membranes were pink with a subtle bluish tint and a capillary refill time of less than one second. Cardiac auscultation revealed a systolic murmur with an intensity of 3 out of 6, with approximately equal intensity on the left and right thoracic walls.

**Figure 2:**
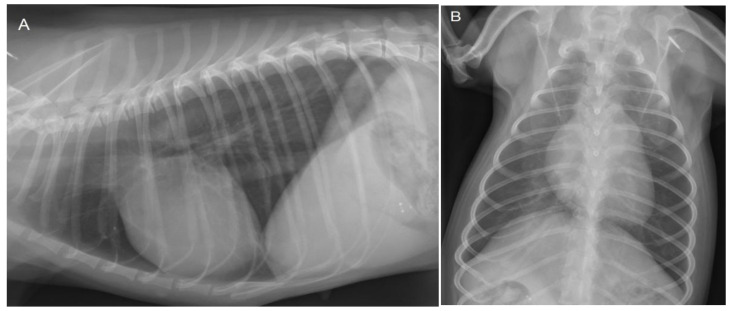
Lateral (**A**) and dorso-ventral (**B**) thoracic radiographs show no abnormalities at 3 years of age. The cardiac silhouette has a normal size and shape in both projections. The vertebral heart scale (VHS) is 10.5, which is within the breed-specific reference range for chihuahuas (8.9–11.0) [14]. There are no changes in the pulmonary parenchyma and pulmonary vasculature visible.

Because of the history of respiratory distress, thoracic radiographs were made, which revealed no abnormalities (Figure 2).

**Figure 3:**
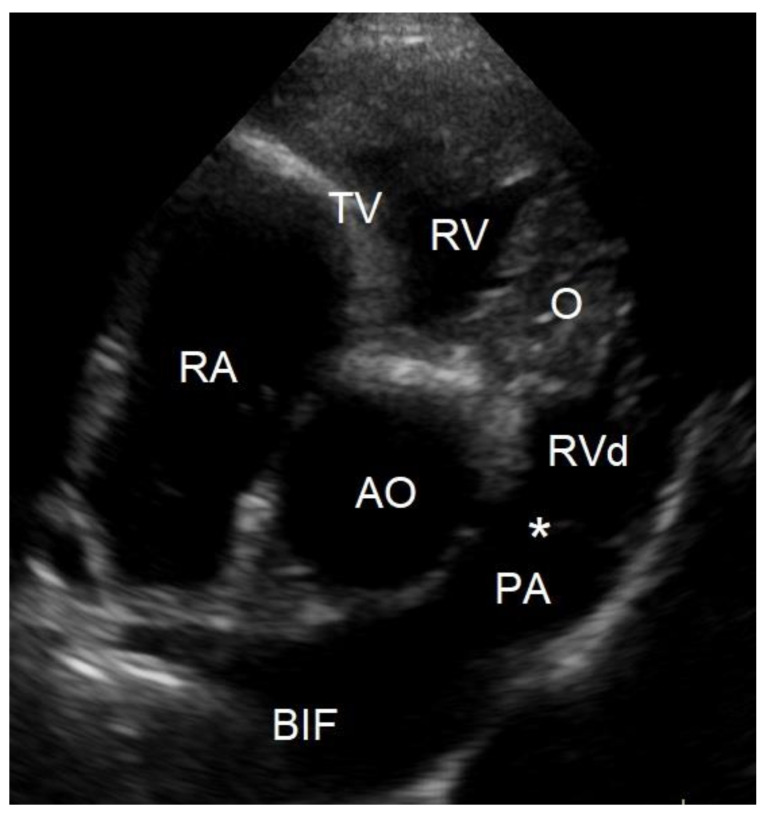
Two-dimensional cross-sectional echocardiographic image at the level of the heart base from the standard right parasternal short axis view at 3 years of age shows a localized muscular hypertrophy in the right ventricle (O). This obstructive muscular lesion resulted in a severe concentric hypertrophy of the right ventricular chamber proximal to the obstruction (RV) as evidenced by a thick wall, compared to the right ventricular chamber distal to the obstruction (RVd), which has a normal wall thickness. AO—aorta, PA—pulmonary artery trunk, *—pulmonic valve, RA—right atrium, TV—tricuspid valve, BIF—pulmonary artery bifurcation.

**Figure 4:**
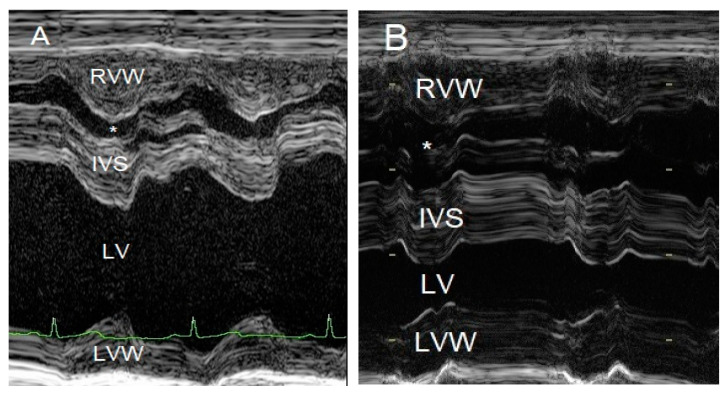
M-mode echocardiographic images of the right and left ventricles at 10 weeks of age (**A**) and 3 years of age (**B**). The most striking difference is the reduced left ventricular chamber (LV) size from normal to too small and the enlargement of the right ventricular chamber (*) caused by a mixed concentric and eccentric hypertrophy. The left ventricular diastolic chamber dimension at 10 weeks of age was 18.7 mm, and this value became 9.9 mm at 3 years of age. Annotations are placed on structures in systole: RVW—right ventricular free wall; IVS—interventricular septum; LVW—left ventricular free wall.

**Figure 5:**
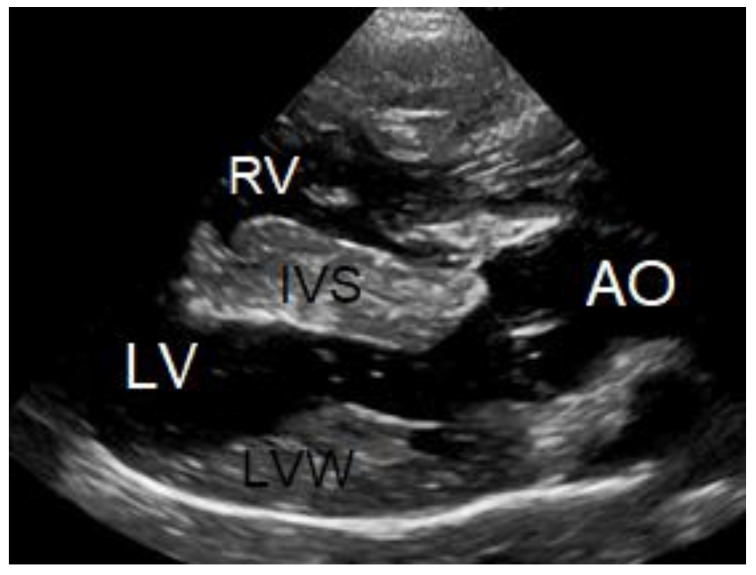
Two-dimensional long axis echocardiographic image from the standard right parasternal long-axis view at 3 years of age shows a positive bubble study, i.e., the appearance of air bubbles, displayed as echogenic dots in the lumen of the left ventricle (LV) after an intravenous injection of 5 ml agitated physiologic saline solution. RV—right ventricle, AO—aorta, IVS—interventricular septum, LVW—left ventricular free wall.

To evaluate the cause of the murmur and the cause of the dyspnea after having obtained thoracic radiographs, an echocardiography was performed in an awake dog without sedation. Echocardiography showed a severe localized right ventricular concentric hypertrophy and a severe obstruction of the right ventricular outflow tract caused by a localized muscular hypertrophy within the right ventricle (Figure 3). The right ventricular lumen at the level of the obstruction measured 1.2 mm, and continuous wave Doppler interrogation revealed a pressure gradient of at least 88 mmHg between the proximal high-pressure and the distal normal-pressure right ventricular compartments. The pulmonary artery and pulmonary valve looked normal, with an annulus diameter of 8 mm and with a normal peak flow velocity of 1.1 m/s (Figure 3). There was no tricuspid valve regurgitation present. The right atrium was subjectively mildly dilated. The left ventricle and left atrium were underfilled (Figure 4). There was a trivial aortic valve regurgitation present, probably secondary to the VSD, since the aortic valve leaflets looked normal. The aortic root diameter was 10 mm. Just underneath the aortic valve, a VSD was seen with a diameter of about 2.5 mm, and color Doppler revealed right-to-left shunting from the high-pressure compartment of the right ventricle to the left ventricle with a pressure gradient of 13 mmHg. A bubble study, i.e., injection of agitated physiologic saline solution intravenously, confirmed the right-to-left shunting at the level of the ventricles (Figure 5). There were no ascites or hepatic venous congestion present.

To evaluate the systemic effect of the intracardiac right-to-left shunting, blood work was performed, which revealed a markedly elevated hematocrit of 0.76 L/L (reference 0.42–0.61 L/L) with a total protein concentration within the reference range.

**Figure 6:**
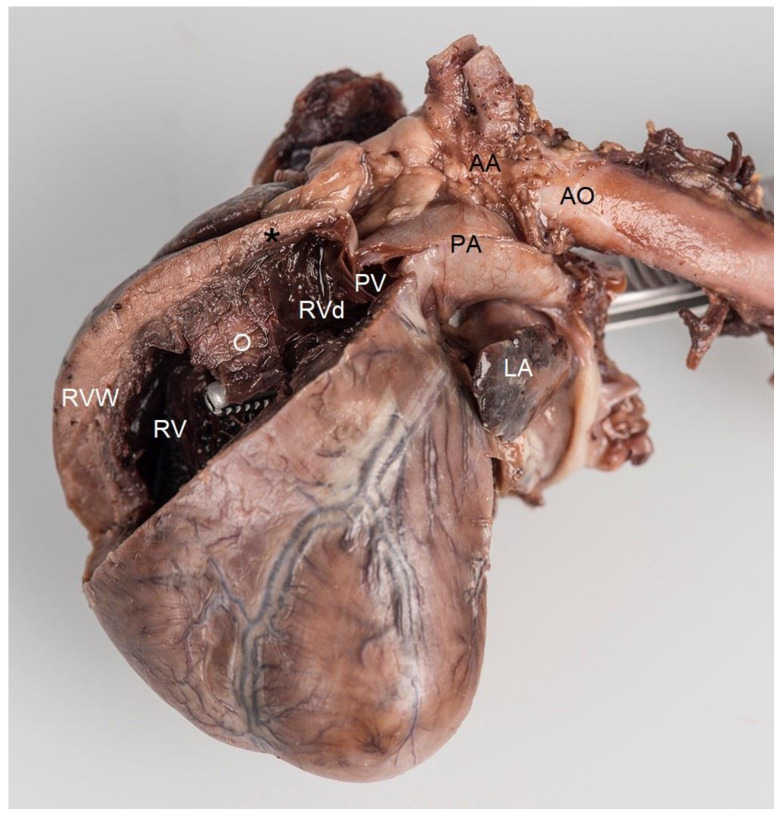
Post-mortem formaldehyde-fixed heart viewed from the left side. The right ventricular free wall was removed to disclose its lumen. Localized muscular hypertrophy in the right ventricular chamber (O) results in a concentric hypertrophy of the right ventricle proximal to the obstruction (RV) as evidenced by a thick right ventricular free wall (RVW), compared to the right ventricular chamber distal to the obstruction (RVd), which has a normal wall thickness (black *). The forceps were introduced from the left ventricle through the ventricular septal defect with its tip in the right ventricle. Note that the tip of the forceps, i.e., the opening of the ventricular septal defect, is very close and proximal to the localized muscular hypertrophy (O). AA—aortic arch, AO—descending aorta, PA—pulmonary artery trunk, PV—pulmonic valve, LA—left auricle.

Because of a poor long-term prognosis, the owner elected for euthanasia, which was performed by the referring veterinarian. The heart was removed from the cadaver, placed in a 10% neutral buffered formalin solution, and returned to the authors’ institution for post-mortem examination. A gross macroscopic investigation of the specimen confirmed the echocardiographic findings. The heart measured 3.0 × 1.5 × 1.0 cm. The right atrial appendage showed a mild dilation (2.0 × 1.0 × 1.0 cm), while the left atrial appendage was very small (1.5 × 1.0 × 0.2 cm). The interventricular septum and both the left and right ventricular free walls measured 5 mm, indicating a moderate right ventricular hypertrophy. A perimembranous ventricular septal defect was noted. In the right ventricle, an atypical muscular bundle of 5 mm in diameter was identified (Figure 6).

**Figure 7:**
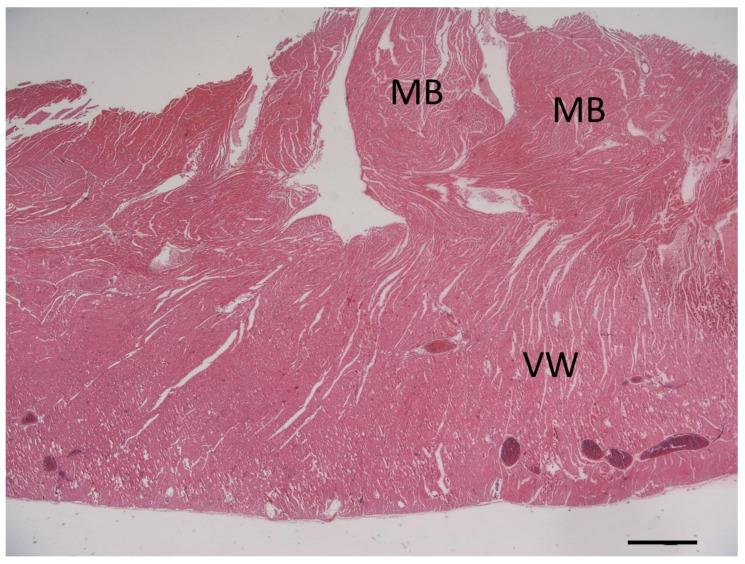
Photomicrograph of the cross-section of the right ventricular wall (VW) at the level of the localized muscular hypertrophy showing whirly arranged cardiomyocytes in the obstructive hypertrophied muscle bundles (MB). Hematoxylin & eosin stain, bar = 1000 µm.

**Figure 8:**
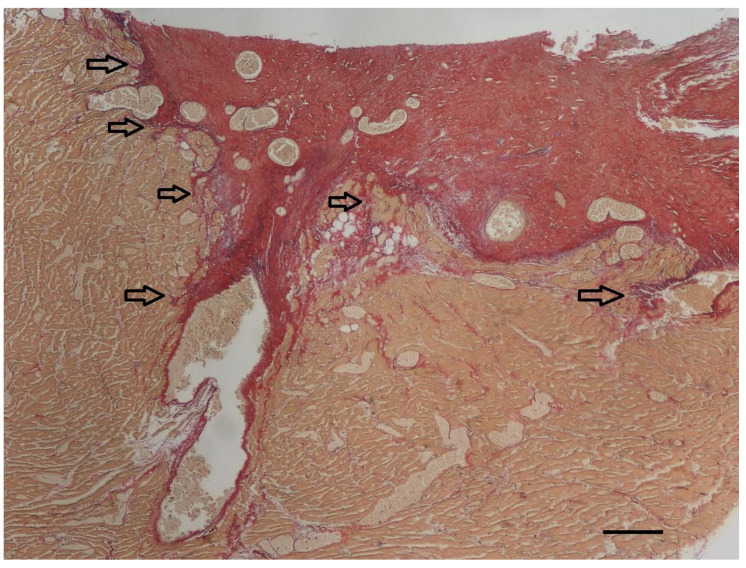
At the endocardial side of the longitudinal section of the right ventricular muscular hypertrophy (yellow), severe focal endocardial fibrosis (red) is present. The arrows indicate infiltration of collagen into the underlying myocardium. In addition to the thick subendocardial layer of collagen, collagen also surrounds the diffuse myocardial bundles throughout the underlying myocardium. Only a minimal increase in elastin fibers (violet) can be appreciated in this specimen accompanying the collagen fibers (red). Modified picrosirius red stain, bar = 200 µm.

Histopathologic examination was performed on representative sites of the heart. The localized right ventricular muscular hypertrophy was composed of whirly arranged cardiomyocytes (Figure 7). Modified picrosirius red stain revealed severe focal endocardial fibrosis at the muscle bundle infiltrating the underlying right ventricular myocardium (Figure 8). The endocardium, myocardium, and pericardium, as well as the great vessels, were normal. The pulmonary alveolar septae showed mild fibrosis but no hemosiderin-loaded macrophages.

## 3. Discussion

In the present case, we report the simultaneous presence of a DCRV and a VSD, also known as the Gasul phenomenon, where the severe right ventricular outflow tract obstruction resulted in right-to-left shunting via the congenital VSD and a subsequent erythrocytosis.

Double-chambered right ventricle is a rare anomaly in dogs and humans [5,6,7,8,9,10,11,12]. 

The exact mechanism of how mid-ventricular septation develops is uncertain. Various theories exist. One suggests that hypertrophy of abnormal septo-parietal trabeculations can lead to DCRV [8]. Another theory suspects that infundibular stenosis is the primary lesion consisting of obstructive fibrous muscle bands at the junction of the main right ventricle and the proximal infundibulum [8]. Because in more than 90% of the humans with DCRV a subaortic VSD is present, the high-pressure jet, i.e., turbulent flow, caused by the left-to-right shunting blood through the VSD is thought to play a role in the genesis of the right ventricular muscular hypertrophy and fibrous tissue formation due to shear stress [7,8]. A recent study in humans showed that the responsible mechanism that contributes to the septation of the right ventricle is an invasive fibroelastic remodeling process of the endocardium into the underlying myocardium through activation of endothelial-to-mesenchymal transition [8]. This mechanism is also a physiologic process described in the embryologic development of heart valves [8]. For the development of DCRV, an underlying anatomical substrate, such as an abnormal congenital muscle bundle, is thought to be required. Turbulent blood flow caused by this bundle and/or flow via a left-to-right shunting VSD can result in shear stress, and in turn localized fibrotic remodeling, and progressive worsening of the right ventricular septation [7,8]. The histologic findings in the presented dog were very similar to those reported in humans, which suggests a similar pathogenesis [8]. In the specimen of the presented dog, histopathology of the abnormal muscular band revealed a large amount of subendocardial fibrosis and infiltration of the underlying myocardium with collagen originating from the surface, similar to that described in humans. Modified picrosirius red stain stains not only collagen but also elastic fibers. In the presented specimen, the elastin content was only minimally increased, and it was not as obvious as it is described in human specimens of DCRV or in hearts with endocardial fibroelastosis [8]. A possible explanation for this finding can be differences in species-specific reaction patterns. Another possible explanation is that endothelial-to-mesenchymal transition in dogs is perhaps not a (major) mechanism in the development of DCRV. Because not every left-to-right shunting VSD results in a DCRV, an underlying substrate in the right ventricle, like an abnormal congenital muscular bundle, is most likely present in patients with the Gasul phenomenon [4,8].

The loud right-sided systolic murmur at 10 weeks of age was caused by the high-velocity jet arising from the left-to-right shunting VSD. However, the systolic murmur at 3 years of age was less intense and equally loud on both sides of the chest. This time the murmur was caused by the severe right ventricular obstruction caused by the DCRV, which anatomically extends to the left side of the thorax. The blood flow velocity via the right-to-left shunting VSD was not high enough to produce turbulent blood flow. In addition, a higher viscosity of the blood, caused by erythrocytosis, reduces the threshold for turbulent flow even further, resulting in a lower-intensity murmur [13].

Erythrocytosis can be relative or absolute [15]. Dehydration, which leads to hemoconcentration, results in not only increased hematocrit but also increased albumin and total protein concentrations. In case of chronic hypoxemia of any cause, renal erythropoietin production causes increased red blood cell production in the bone marrow [15]. Primary erythrocytosis (polycythemia vera) and erythrocytosis resulting from a paraneoplastic syndrome (i.e., renal malignant lymphoma) are not associated with hypoxemia [15]. To confirm hypoxemia, arterial blood gas analysis is the first choice of test. However, diagnostic imaging findings (using either color Doppler echocardiography, bubble-study, or angiocardiography) showing right-to-left intra- or extra-cardiac shunting can indirectly confirm hypoxemia, if obtaining an arterial blood sample was unsuccessful [15].

Because DCRV is a rare condition, there are only a few papers that report on the associated clinical signs [9,10,11,12]. Clinical presentation in dogs and humans is very similar and can range from lack of clinical signs through dyspnea on exertion to congestive right-sided heart failure [7,8,9,10,11,12]. Most clinical signs reported in dogs are either exercise intolerance with or without syncope because of low cardiac output secondary to the right ventricular obstruction or signs of congestive right-sided heart failure [9,10,11,12]. Because the reported pressure gradients caused by the DCRV both in dogs and humans are typically less than 80 mmHg, the right ventricular systolic pressure rarely exceeds left ventricular systolic pressure [7,8,9,10,11,12]. Flow-direction through the VSD in these cases is left-to-right [7,8,9,10,11,12]. In the present case, at 10 weeks of age, the right ventricular obstruction was only mild, which allowed left-to-right shunting via the VSD. However, this high-velocity turbulent jet resulted in structural changes in the right ventricle that led to a roughly 60 mmHg increase in the right ventricular obstruction in 3 years, resulting in a supra-systemic right ventricular systolic pressure and, in turn, a flow-reversal via the VSD. The leading clinical sign in the presented dog was exertional dyspnea, most likely attributable to systemic hypoxemia due to the shunting of deoxygenated blood from the right ventricle to the left ventricle through the VSD [1,2,3,9]. The initial diagnostic test of choice in a dog presenting with dyspnea without noisy breathing is usually thoracic radiography. In the present case, no radiographic abnormalities were seen in the thorax, which finding made most pulmonary parenchymal pathologies, including cardiogenic pulmonary edema, as the cause of dyspnea very unlikely. The cardiac silhouette was normal in size. Concentric right ventricular hypertrophy would not result in cardiac chamber enlargement, especially when the left ventricle is underfilled. As a result, radiographic cardiomegaly is unlikely, similar to cases of severe pulmonic stenosis. Echocardiography with a bubble-study confirmed intra-cardiac right-to-left shunting as the most likely cause of the leading clinical signs. Arterial blood gas analysis was not performed because of difficulties obtaining an arterial blood sample in such a small dog. Exercise intolerance was most likely caused by insufficient tissue oxygenation due to impaired tissue perfusion caused by erythrocytosis and hypoxemia due to right-to-left intra-cardiac shunting. Both cerebral hypoxia and cerebral hypotension can lead to transient loss of consciousness, which was considered the most likely reason for the syncopal episodes in this dog.

Surgical resection of the obstructive muscular band generally offers a favorable long-term prognosis in humans without recidivating the lesion for the long term [8]. Though surgical therapy of DCRV has been reported in dogs [10,11], open heart surgery for the presented dog would have been a very high-risk procedure due to its small size. Catheter-based intervention using a cutting balloon followed by a high-pressure balloon dilation has been reported in a dog, but after an initial improvement, the procedure failed to result in long-term success [12]. For these reasons, no surgical or catheter-based interventional therapy was offered to the owner. Palliative long-term management of the presented dog would have focused on reducing the hematocrit by repeated phlebotomy or medical leech phlebotomy [13]. As this therapy cannot offer a cure and must be performed repeatedly, the owner elected for euthanasia because of the severe clinical signs.

## 4. Conclusions

We present a dog with progressive obstruction to blood flow within the right ventricle due to septation of the right ventricle, resulting in a progressive increase in right ventricular systolic pressure and shunt reversal via a congenital VSD, with subsequent erythrocytosis, which in turn prompted the appearance of clinical signs. Histologic findings of the right ventricular obstructive lesion suggest the same pathogenesis of DCRV as is suspected in humans, i.e., endothelial-to-mesenchymal transition caused by turbulent blood flow in the right ventricle arising from the initially present left-to-right shunting VSD.

## Data Availability

Not applicable.
